# Treatment of bilateral popliteal artery aneurysms

**DOI:** 10.1590/1677-5449.180142

**Published:** 2019-11-29

**Authors:** José Aderval Aragão, Fabio Guilherme Gonçalves de Miranda, Iapunira Catarina Sant’Anna Aragão, Felipe Matheus Sant’Anna Aragão, Francisco Prado Reis

**Affiliations:** 1 Universidade Federal de Sergipe – UFS, Aracaju, SE, Brasil.; 2 Universidade Tiradentes – UNIT, Aracaju, SE, Brasil.; 3 Fundação Beneficência Hospital Cirurgia, Serviço de Cirurgia Vascular, Aracaju, SE, Brasil.; 4 Centro Universitário de Volta Redonda – UNIFOA, Volta Redonda, RJ, Brasil.

**Keywords:** aneurysm, popliteal artery, peripheral arterial disease, peripheral vascular diseases, chronic disease, hypertension, vascular surgical procedures

## Abstract

Popliteal artery aneurysms are the most frequent type of peripheral aneurysm, accounting for 85% of the all of these aneurysms. Usually asymptomatic, they are generally diagnosed during clinical examination. Incidence is higher among males and seniors. They are bilateral in 50% of the cases and 60% are associated with abdominal aortic aneurysms. This paper describes a 72-year-old male patient who presented with two bilateral pulsatile masses, one in each popliteal region, was otherwise asymptomatic, and had a history of hypertension and dyslipidemia. Clinical examination and ultrasound imaging confirmed a diagnosis of bilateral aneurysms of the popliteal arteries. Popliteal artery aneurysms can be treated with open bypass surgery, with or without aneurysm resection, or with endovascular surgery. This Therapeutic Challenge discusses these possibilities.

## INTRODUCTION

Popliteal artery aneurysms account for approximately 85% of all peripheral arterial aneurysms and are bilateral in 50% of patients.^1^^,^ 2 They are more common among males and the elderly.^3^ Among younger patients, a relationship has been observed with osteochondroma.^4^^,^ 5 In the majority of cases, these aneurysms are asymptomatic, although as volume increases they can cause pain and edema due to compression of nerves and veins.^6^

Popliteal artery aneurysms rarely rupture and their most significant complications are thrombosis and embolization.^7^ Treatment is surgical, which can be accomplished using endovascular procedures or open surgery (interposition or bypass with the great saphenous vein reversed or a prosthetic graft), with or without resection of the aneurysm.^8^^-^ 10 This Therapeutic Challenge will discuss these possibilities.

### Part I – Clinical situation

**Figure 1:**
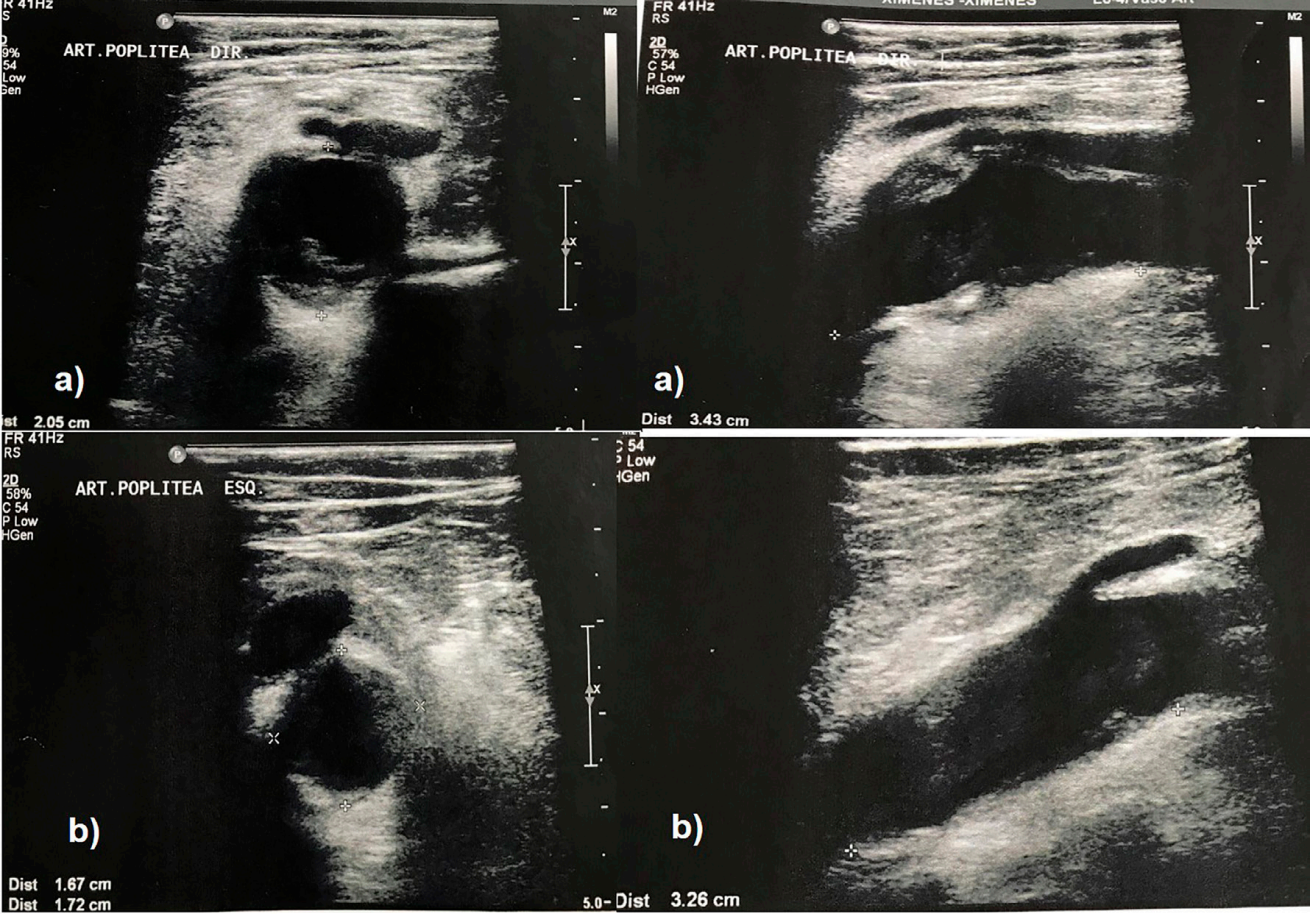
Aneurysmal dilatation of the right (a) and left (b) popliteal arteries, with mural thrombus visible on ultrasound.

The patient was a 72-year-old male who presented with bilateral popliteal artery aneurysms, was otherwise asymptomatic, and had a history of arterial hypertension and dyslipidemia. On physical examination there were palpable pulsatile masses suggestive of aneurysms in both popliteal regions, with no thrill or murmur in either limb. Dorsal pedal and posterior tibial pulses were palpable and the ankle-brachial index at rest was normal on both sides. No signs of ischemia were observed and no other vascular disorders were found in the lower limbs. Duplex scanning of the lower limbs revealed two popliteal artery aneurysms: one on the right measuring approximately 2.05 cm at its maximum diameter and 3.43 cm in length (Figure 1A), and the other on the left, with a maximum diameter of 1.72 cm and length of 3.26 cm (Figure 1B). There are a number of therapeutic possibilities in such a situation:

1- Endovascular procedure;
2- Open surgery via medial access with interposition or bypass using the great saphenous vein or prosthetic graft, with or without resection of the aneurysm sac;
3- Open surgery via posterior access with interposition of the great saphenous vein or prosthetic graft, with or without resection of the aneurysm sac.

### Part II – What was done

**Figure 2:**
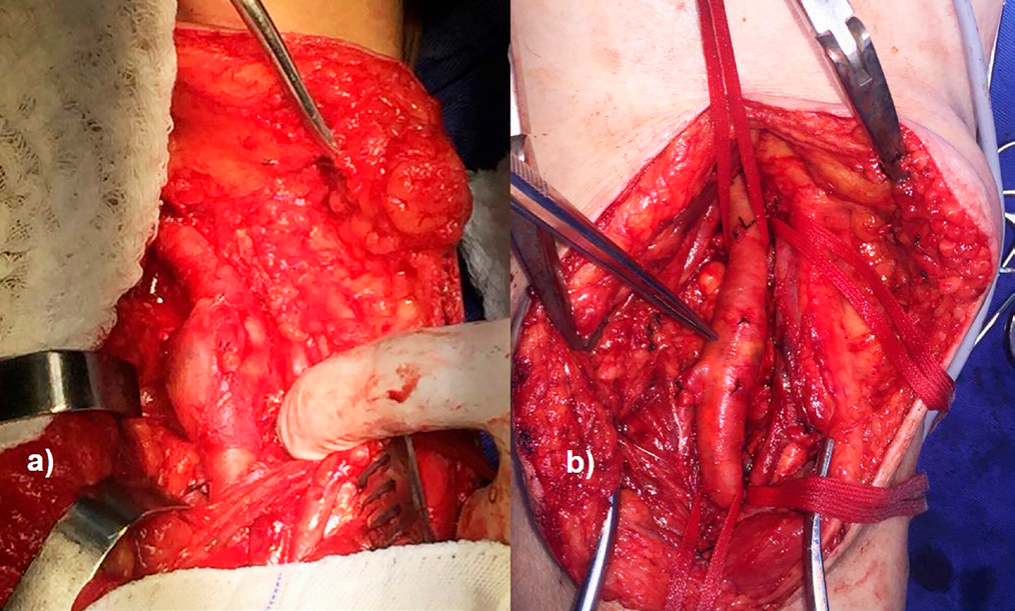
Surgical exposure of popliteal artery aneurysms, a saccular aneurysm on the right (a) and a fusiform aneurysm on the left (b).

**Figure 3:**
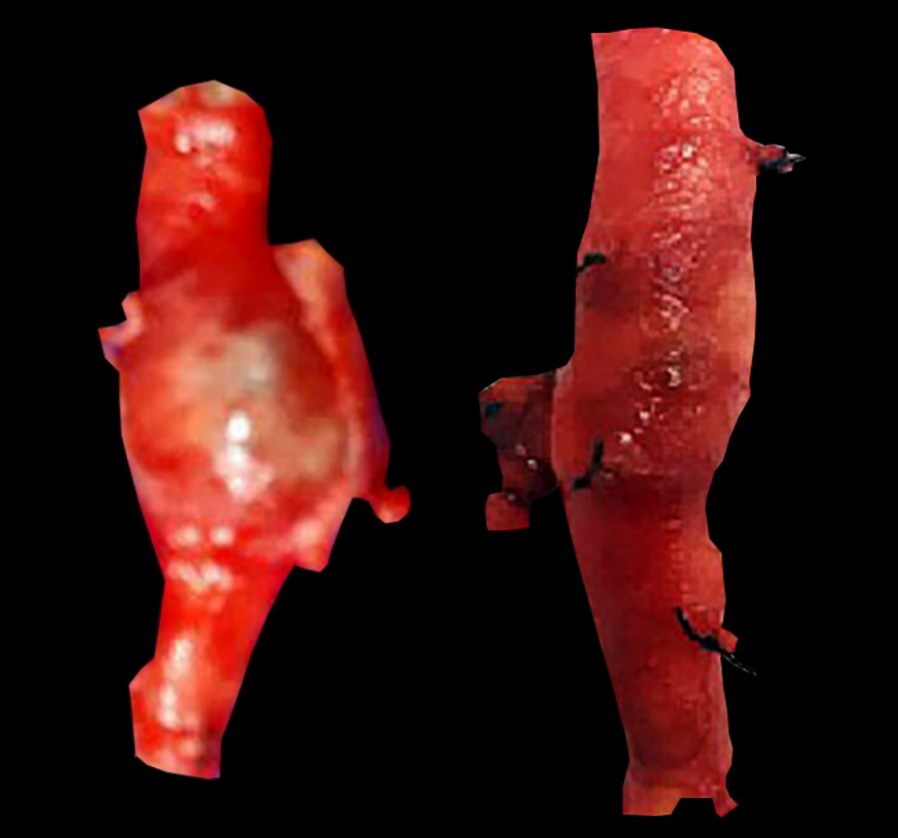
Surgical resection of a popliteal artery aneurysm.

**Figure 4:**
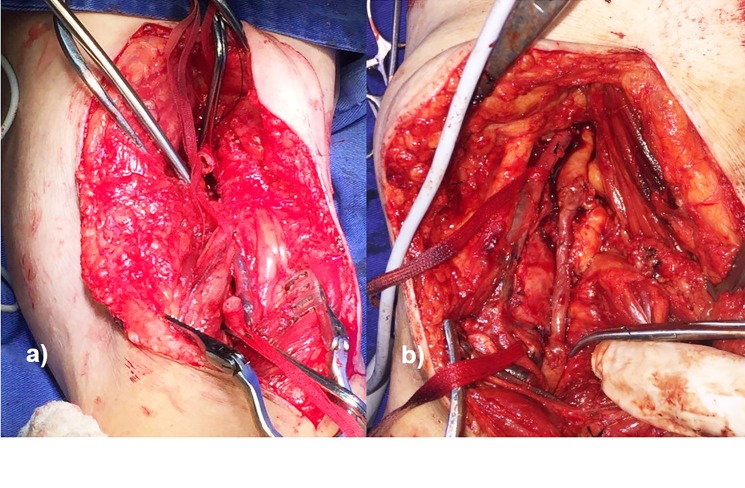
Exposure of the proximal and distal stumps (a) and popliteal-popliteal interposition of the reversed great saphenous vein (b).

Under epidural anesthesia, a surgical procedure to resect the aneurysm was performed in each lower limb, with a 90-day interval. The popliteal fossae were approached via a longitudinal, S-shaped incision through the skin and subcutaneous tissue. After dissection and exposure of the popliteal artery aneurysms (Figure 2), the proximal and distal portions were repaired and clamped and the isolated stumps were sectioned, followed by resection of the PAAs (Figure 3) and interposition of the reversed great saphenous veins in the popliteal-popliteal segment (Figure 4).

## DISCUSSION

Popliteal artery aneurysms are the most common type of peripheral aneurysm, in 50% of cases they present bilaterally, and they can be found coexisting with abdominal aortic aneurysms in 60% of cases.^2^ Although 80% of them are asymptomatic at the time of diagnosis,^11^ they tend to become symptomatic over time, at a rate of 14% per year.^12^ In contrast with aneurysms of the abdominal aorta, in which rupture is the major complication, popliteal artery aneurysms are prone to thrombosis with acute ischemia and risk of limb loss.^13^^,^ 14 Popliteal aneurysms are often asymptomatic and in general diagnosis is made by physical examination, by palpation of a wide arterial pulse in the popliteal fossa and, incidentally, by imaging exams (ultrasound, angiotomography, and magnetic resonance), which are also used to complement diagnosis and improve surgery planning, primarily when endovascular repair will be attempted.^9^ At our service, we normally use ultrasound as the method of choice for anatomic studies of the popliteal artery, because it is a cheaper method that is noninvasive and can be repeated easily, without harm to the patient.^15^^,^ 16 However, the reliability of ultrasound measurements is examiner dependent.^17^^,^ 18 The majority of vascular surgeons indicate surgery for PAAs with diameters greater than or equal to 2.0 cm.

The classic treatment for a PAA consists of exclusion of the aneurysm with a bypass using an autologous or synthetic graft.^19^ The technique most often used is via a medial access with exclusion achieved by ligature of the popliteal artery upstream and downstream of the aneurysm, followed by popliteal -popliteal bypass with the great saphenous vein either reversed or devalved or with Dacron or PTFE grafts.

The advantages of this technique are its simplicity and reduced likelihood of trauma or iatrogenic injuries. Additionally, the saphenous vein can be accessed via the same incision. Disadvantages include maintenance of the thrombosed aneurysmal mass and patency of its localized branches, which may not entirely exclude the PAA. It is possible to entirely expose and open the PAA via this access, but in order to do so via this route of exposure it is necessary to section tendons and muscles at the level of the knee (semitendinosus, semimembranosus, gracilis, and gastrocnemius). The advantages of this larger exposure would be the possibility of removal of thrombi or of the aneurysm itself, of internal ligature of the branches, and of decompression of neighboring structures.

Another method (the one used in this case) is to use a posterior access with resection or opening of the aneurysm (similar to with an aortic aneurysm), ligature of the branches and interposition of a continues autologous or synthetic graft. The drawback of this access is the limited scope for access to the popliteal vessels and the possibility of injuring the fibular nerve.^20^ A saphenous vein with good caliber is the ideal graft material in these cases, since it is autologous, more malleable, more resistant to folding and to thrombosis, and less prone to infections. In the present case, surgical access to the aneurysms in both limbs was accomplished via a posterior route, which is usually considered preferable in cases with short aneurysms limited to the popliteal fossa. This procedure is as described by Pulli et al.,^21^ who also employed this type of posterior approach to aneurysms limited to the popliteal fossa. According to Wagenhäuser et al.,^10^ surgical access to a popliteal artery aneurysm can be achieved via medial or posterior routes and there is no significant difference in the long-term results.

Open surgical repair of aneurysms of the popliteal artery is a safe procedure with low rates of perioperative complications and excellent long term rates of both graft patency and limb salvage, particularly in asymptomatic cases.^21^ In the present case, there were no intercurrent conditions during the first 10 first months of follow-up.

Over recent years, endovascular exclusion of popliteal artery aneurysms has emerged as a new weapon in the arsenal of vascular surgery procedures available to vascular surgeons.^22^ This treatment option has grown in importance, especially for patients who have a high surgical risk or when no saphenous vein or prosthesis is available for grafting.^9^ Endovascular approaches are being used with increasing frequency as techniques and materials improve and because of their lower invasivity. This procedure is limited by the position of the stent, very often crossing the knee joint, which makes fatigue and fracture of the metallic material more likely. However, development of more flexible self-expanding stents has reaped more promising results. The results of the procedure are better in cases with good anatomy and at least two patent distal arteries.^23^

According to von Stumm et al.,^24^ over the last two decades endovascular repair of popliteal artery aneurysms has proven comparable to open surgery over the medium term and it appears to be a safe alternative to conventional open surgical repair. However, Wagenhäuser et al.^10^ have concluded that open surgical repair remains the gold standard. Notwithstanding, endovascular repair has been performed with acceptable results in relation to open surgery. Comparative studies have shown primary patency rates in the range of 86.6 to 95.0% for endovascular techniques and 78.8 to 87.5% for open surgery using the saphenous vein as graft.^25^ However, the 30 and 90-day reintervention rates after the initial endovascular procedure are considerably higher than after open surgery.^24^

## CONCLUSIONS

A review of the literature suggests that open surgical treatment of PAAs has similar patency rates to endovascular repair, with slightly higher surgical complication rates, although randomized comparative studies are limited. In the case described here, elective surgical intervention in a patient with low surgical risk and good life expectancy was a lasting therapeutic strategy that is appropriate and safe and achieves good initial and long-term results.
