# Supplementary material for: Treatment of bilateral popliteal artery aneurysms
Source: J Vasc Bras. 2019 Nov 29;19:e20180142. doi: 10.1590/1677-5449.180142 (PMC6900880; doi:10.1590/1677-5449.180142)
Supplement: Translation

# Translation: Tratamento de aneurisma de artéria poplítea bilateral

**Authors:** José Aderval Aragão, Fabio Guilherme Gonçalves de Miranda, Iapunira Catarina Sant’Anna Aragão, Felipe Matheus Sant’Anna Aragão, Francisco Prado Reis

**Affiliations:** 1 Universidade Federal de Sergipe – UFS, Aracaju, SE, Brasil.; 2 Universidade Tiradentes – UNIT, Aracaju, SE, Brasil.; 3 Fundação Beneficência Hospital Cirurgia, Serviço de Cirurgia Vascular, Aracaju, SE, Brasil.; 4 Centro Universitário de Volta Redonda – UNIFOA, Volta Redonda, RJ, Brasil.

**Keywords:** aneurisma, artéria poplítea, doença arterial periférica, doenças vasculares periféricas, doença crônica, hipertensão arterial sistêmica, procedimentos cirúrgicos vasculares

## Abstract

O aneurisma de artéria poplítea (AAP) é o mais frequente dos aneurismas periféricos, correspondendo a 85% do total de aneurismas. Normalmente, são assintomáticos, e o diagnóstico, em geral, é feito por meio do exame físico, tendo uma maior incidência no sexo masculino e em idosos. São bilaterais em 50% dos casos e, em 60% das vezes, estão associados a aneurisma de aorta abdominal. Apresentamos um paciente do sexo masculino com 72 anos de idade, com duas massas pulsáteis em ambas as regiões poplíteas, assintomático, com história de hipertensão arterial e dislipidemia. Os exames clínico e de imagem ultrassonográfica confirmaram o diagnóstico de aneurisma bilateral de artéria poplítea. Os AAP podem ser operados por meio de derivações por cirurgia aberta, com ou sem resseção do aneurisma ou por meio de cirurgia endovascular. Esse desafio discute estas possibilidades.

## INTRODUÇÃO

O aneurisma de artéria poplítea corresponde a aproximadamente 85% dos aneurismas arteriais periféricos, sendo bilateral em 50% dos pacientes^1^^,^ 2. São mais comuns no sexo masculino e em idosos^3^. Já em pacientes jovens, tem sido observada uma relação com osteocondroma^4^^,^ 5. Na maioria dos casos, o aneurisma é assintomático, podendo, com o aumento de volume, apresentar dor e edema devido à compressão de nervos e das veias^6^.

O aneurisma de artéria poplítea dificilmente se rompe, porém tem como principais complicações a trombose e a embolização^7^. O tratamento é cirúrgico, podendo ser realizado por meio de procedimento endovascular ou cirurgia aberta (interposição ou ponte com veia safena magna invertida ou com enxerto protético), com ou sem resseção do aneurisma^8^^-^ 10. Esse desafio discute essas possibilidades.

### Parte I – Situação clínica

Paciente do sexo masculino, com 72 anos de idade, apresentando aneurisma bilateral de artéria poplítea, assintomático, com história de hipertensão arterial e dislipidemia. Ao exame físico, foi possível palpar a presença de massas pulsáteis bilaterais, sugestivas de aneurismas, sem frêmito ou sopro em ambas regiões poplíteas. Os pulsos dorsais dos pés e tibiais posteriores foram palpáveis, e o índice tornozelo-braquial em repouso foi normal em ambas as extremidades. Não foi observado sinal de isquemia, nem a detecção de outras alterações vasculares nas demais regiões dos membros inferiores. O exame de *duplex scan* de membros inferiores mostrou a presença de dois aneurismas das artérias poplíteas: um à direita, medindo aproximadamente 2,05 cm de diâmetro máximo e 3,43 cm de extensão (Figura 1), e outro à esquerda, com 1,72 cm de diâmetro máximo e 3,26 cm de extensão (Figura 1). Diante dessa situação, havia algumas possibilidades terapêuticas:

**Figura 1:** Dilatação aneurismática da artéria poplítea direita (a) e esquerda (b) com presença de trombo mural vista pelo ultrassom.

1- Procedimento endovascular;
2- Cirurgia aberta (via medial com interposição ou ponte com veia safena magna ou com enxerto protético), com ou sem resseção do saco aneurismático;
3- Cirurgia aberta via posterior com interposição de veia safena magna ou com enxerto protético, com ou sem resseção do saco aneurismático.

### Parte II – O que foi feito

Sob anestesia peridural, o procedimento cirúrgico de ressecção do aneurisma foi realizado em cada membro inferior, com intervalo de 90 dias. As regiões dos cavos poplíteos foram abordadas por meio de uma incisão longitudinal em forma de S que abrangeu pele e tecido subcutâneo. Após dissecção e exposição dos aneurismas das artérias poplíteas (Figura 2), suas porções proximais e distais foram reparadas e pinçadas com clampes, além de terem sido seccionadas e seus cotos isolados, seguido pela ressecção (remoção cirúrgica) dos AAPs (Figura 3) e interposição em continuidade com veias safenas magnas invertidas no segmento poplíteo-poplíteo (Figura 4).

**Figura 2:** Exposição cirúrgica dos aneurismas das artérias poplíteas, sendo um sacular à direita (a) e outro fusiforme à esquerda (b).

**Figura 3:** Ressecção cirúrgica de aneurisma das artérias poplíteas.

**Figura 4:** Exposição dos cotos proximal e distal (a) e interposição de veia safena magna invertida poplíteo-poplíteo (b).

## DISCUSSÃO

O aneurisma da artéria poplítea é o mais comum dos aneurismas periféricos e se apresenta bilateralmente em 50% dos casos, podendo também coexistir com o aneurisma da aorta abdominal em 60% dos casos^2^. Embora 80% deles sejam assintomáticos no momento do diagnóstico^11^, eles vão se tornando sintomáticos com o tempo, a uma taxa de 14% ao ano^12^. Diferentemente dos aneurismas da aorta abdominal, que têm a rotura como maior complicação, os aneurismas da artéria poplítea são caracterizados pela trombose, com isquemia aguda e risco de perda de membro^13^^,^ 14. Os aneurismas poplíteos são frequentemente assintomáticos, e o diagnóstico, em geral, é feito por meio do exame físico, da palpação de pulso arterial amplo na região do cavo poplíteo e, casualmente, por meio de exame de imagem (ultrassom, angiotomografia e ressonância magnética), os quais são usados também para complementação diagnóstica e melhor planejamento cirúrgico, principalmente nos reparos por via endovascular^9^. Em nosso serviço, temos utilizado normalmente o ultrassom como método de escolha para caracterização anatômica da artéria poplítea, por ser um método mais barato, não invasivo, e que pode ser repetido facilmente sem prejudicar o paciente^15^^,^ 16. No entanto, a confiabilidade das medidas de ultrassom é examinador dependente^17^^,^ 18. A maioria dos cirurgiões vasculares indica cirurgia para AAP com diâmetro igual ou maior que 2,0 cm.

O tratamento clássico do AAP consiste na exclusão do aneurisma com *bypass* por meio de uma prótese autóloga ou sintética^19^. A técnica mais usada é o acesso medial com exclusão feita por meio de ligadura da artéria poplítea a montante e a jusante do aneurisma, seguido de bypass de veia safena magna invertida ou devalvulada poplíteo-poplíteo ou com prótese de Dacron ou PTFE.

A vantagem dessa técnica seria sua simplicidade e menores chances de trauma e de lesões iatrogências. Além disso, a veia safena ficaria acessível pela mesma incisão. Como desvantagem, deve-se considerar a manutenção da massa aneurismática trombosada ou perviedade dos seus ramos locais, o que, eventualmente, pode não excluir totalmente o AAP. O AAP pode ser inteiramente exposto e aberto por essa via, mas, para isso, essa via de exposição exige seção de tendões e musculaturas ao nível do joelho (semitendinoso, semimembranoso, grácil, gastrocnemio). As vantagens dessa exposição mais ampla seriam a possibilidade de remoção de trombos ou do próprio aneurisma, de ligadura interna dos ramos e descompressão das estruturas vizinhas.

Outra forma (adotada no presente caso) é a via posterior com ressecção ou abertura do aneurisma (similar ao aneurisma aórtico), com ligadura dos ramos e com interposição de enxerto em continuidade material autólogo ou sintético. O percalço dessa via seria a limitação ao acesso mais amplo aos vasos poplíteos e a possibilidade de lesão do nervo fibular^20^. A veia safena com bom calibre é o enxerto ideal nesses casos, por ser material autólogo, mais maleável, mais resistente a dobras e à trombose e menos sujeito a infeções. No presente caso, a abordagem cirúrgica dos aneurismas em ambos os membros foi realizada por via posterior, que usualmente tem sido considerada como a preferida em casos de aneurismas curtos e limitados ao cavo poplíteo. Esse procedimento está de acordo com o trabalho de Pulli et al.^21^, que também realizaram esse tipo de abordagem posterior para os aneurismas limitados ao cavo poplíteo. Para Wagenhäuser et al.^10^, o acesso cirúrgico do aneurisma de artéria poplítea pode ser feito por via medial ou posterior, não havendo diferença significativa nos resultados obtidos em longo prazo.

O reparo cirúrgico aberto dos aneurismas de artéria poplítea é um procedimento seguro, com baixas taxas de complicações perioperatórias, e mantém excelentes taxas de perviedade ao enxerto e ausência de amputação em longo prazo, particularmente em lesões assintomáticas^21^. No presente caso, não houve intercorrências durante os 10 primeiros meses de seguimento.

Nos últimos anos, a exclusão de aneurismas de artéria poplítea por via endovascular tem representado uma nova ferramenta no arsenal de procedimentos da cirurgia vascular e dos cirurgiões vasculares^22^. Essa opção de tratamento tem ganhado importância, em especial, para aqueles pacientes que apresentam alto risco cirúrgico, indisponibilidade de veia safena ou prótese para a confecção do enxerto^9^. A abordagem endovascular vem sendo feita com crescente frequência devido à melhora na técnica e nos materiais e menor invasividade. Esse procedimento tem como limitação a implantação de *stent*, muitas vezes atravessando a articulação do joelho, o que favorece a fadiga e a fratura do material metálico. Porém, o desenvolvimento de *stents* autoexpansíveis mais flexíveis tem proporcionado resultados mais promissores. Esse procedimento tem melhor resultado para casos com boa anatomia e pelo menos duas artérias distais pérvias^23^.

Para von Stumm et al.^24^, o reparo endovascular do aneurisma da artéria poplítea, ao longo das duas últimas décadas, tem se mostrado, em médio prazo, comparável à cirurgia aberta e parece ser uma alternativa segura ao reparo cirúrgico aberto convencional. Porém, Wagenhäuser et al.^10^ concluíram que o reparo cirúrgico aberto continua a ser o padrão-ouro. Por outro lado, o reparo endovascular tem sido realizado com resultados aceitáveis em relação à cirurgia aberta. Estudos comparativos têm mostrado taxas de perviedade primária de 86,6 a 95,0% e de 78,8 a 87,5%, respectivamente, para técnicas endovasculares e abertas com veia safena como enxerto^25^. Porém, as taxas de reintervenção durante 30 e 90 dias após o procedimento endovascular inicial têm sido consideravelmente maiores do que após a cirurgia aberta^24^.

## CONCLUSÃO

A revisão da literatura sugere que os tratamentos cirúrgicos abertos dos AAP têm resultado de perviedade similar ao endovascular, com taxas de complicações cirúrgicas um pouco maiores, embora os trabalhos randomizados comparativos sejam limitados. No presente caso, a intervenção cirúrgica eletiva, realizada em paciente com baixo risco cirúrgico e boa expectativa de vida, constituiu-se em uma estratégia terapêutica duradoura, adequada, segura e com bons resultados iniciais e em longo prazo.
